# Development and characterization of PAN/GO-tyrosine hollow fiber membranes for enhanced heavy metal adsorption and SPME-spectrophotometric detection†

**DOI:** 10.1039/d4ra08423c

**Published:** 2025-02-05

**Authors:** Maryam Abbasi Tarighat, Fatemeh Barghandan, Seyed Abdollatif Hashemifard, Gholamreza Abdi

**Affiliations:** a Deparmen of Chemistry, Faculty of Nano and Bio Science and Technology, Persian Gulf University Bushehr 75169 Iran matarighat@pgu.ac.ir matarighat@gmail.com; b Sustainable Membrane Technology Research Group, Faculty of Petroleum, Gas and Petrochemical Engineering, Persian Gulf University Bushehr 75169 Iran; c Department of Biotechnology, Persian Gulf Research Institute, Persian Gulf University Bushehr 75169 Iran

## Abstract

PAN-based fiber membranes have attracted significant attention owing to their potential in heavy metal removal. However, they show low selectivity, poor hydrophilicity, and low adsorption efficiency, which limits their performance. In the present investigation, PAN/GO-Tyr hollow fiber (PAN/GO-Tyr HF) membranes were fabricated *via* the phase inversion of PAN/GO, and subsequent grafting of tyrosine was done *via* self-polymerization to the surface of the membrane. FESEM characterization showed significant changes in the morphology of PAN/GO-Tyr HF, such as the presence of thumb-like pores in the outer layer of the membrane. AFM results of PAN/GO-Tyr HF revealed a more homogeneous surface with low roughness. Results of derivative and cumulative BJH adsorption showed that micropores in the structure of PAN/GO-Tyr HF were in the size range below 20 Å, while PAN/GO HF had a higher percentage of mesopores with larger pores. Furthermore, BET analysis showed that PAN/GO-Tyr HF had more complex pores, greater surface area, and larger hysteresis behavior. A detailed assessment was conducted on the impact of surface alterations on several adsorption processes as well as adsorption kinetics characteristics. Adsorption kinetics fitted better with the pseudo-second order model and confirmed multiple layer adsorption mechanism and chemisorption process. Finally, the fabricated hollow fiber was used as an adsorbent for SPME-spectrophotometric detection. The adsorbent demonstrated good linearity and correlation coefficients over the concentration ranges of 0.005–4.0 ppb (*r*^2^ = 0.989), 0.01–10.0 ppb (*r*^2^ = 0.974), 0.003–2.0 ppb (*r*^2^ = 0.999) and 0.05–1.0 ppb (*r*^2^ = 0.989) and detection limits of 0.004, 0.005, 0.002 and 0.01 ppb for As^3+^, Cu^2+^, Sn^2+^ and Pb^2+^, respectively. Furthermore, reusability of the membrane was evaluated for six adsorption–desorption cycles.

## Abstract 2

PAN-based fiber membranes have attracted significant attention owing to their potential in heavy metal removal.

## Graphical abstract

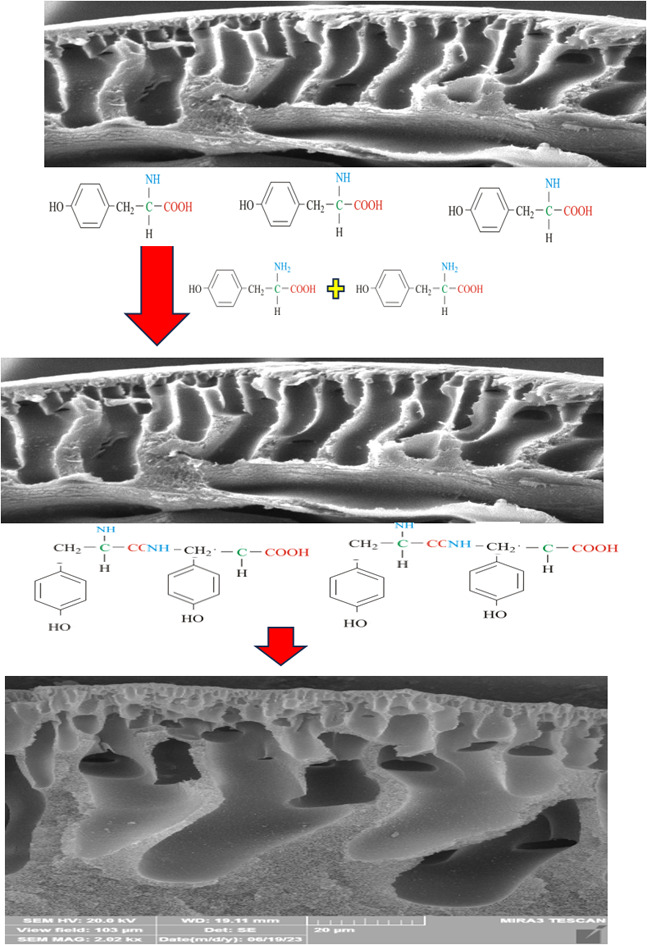

## 1. Introduction

Heavy metals are well-known environmental contaminants owing to their toxicity, extended atmospheric half-lives, and capacity for bioaccumulation inside the human body. The contamination of water and aquatic ecosystems by hazardous heavy metals is a significant environmental issue that has an impact on human health. Because heavy metals may affect people and other organisms that are exposed to them through the food chain by combining with different environmental factors, including water, soil, and air, they can become extremely dangerous.

Both soil and water contain arsenic, which is the 20th most prevalent naturally occurring metal in the Earth's crust. Because of its extreme toxicity, it poses a major risk to living organisms. In general, this element can adversely affect the neurological, pulmonary, and circulatory systems. In particular, it poses an acute risk of bladder, lung, and skin cancers, even when exposed to trace amounts. The toxicity of arsenic has been observed in plants as well as mammals. Mercury is another very hazardous heavy element. Once released into the environment, mercury compounds (fungicides, paints, tattoo colors) gradually find their way into rivers, seawater, and soil. Tin may enter the body through the mouth, respiratory system, or skin and can have harmful effects on the liver, chromosomes, and brain.^1,2^ The burning of fossil fuels, mining, and other human activities are all contributing to the steady rise in atmospheric lead levels. When exposed to higher than ideal levels, lead is harmful to human health. It is known that copper is an essential micronutrient for every living organism. It is involved in the production of chlorophyll, photosynthesis, and the metabolism of carbohydrates and proteins, among other regular physiological processes of plants. Important metabolic pathways are altered by copper deficiency; however, increased exposure to it results in toxicity.

During the past few decades, several spectrometric,^3–8^ voltametric,^9,10^ chromatographic^11,12^ and chromatography-MS^2,13^ methods have been proposed for the detection and determination of metal ions. Due to the low concentration of heavy metals in most real samples as well as the complexity of sample matrices, before measurement, a suitable pre-treatment step should be performed wherein analytes are concentrated and also separated from the interferences in the sample.^14^

Solid-phase extraction (SPE) and liquid–liquid extraction (LLE) methods have been used since the distant past for removing heavy metals as well as other pollutants from various matrices. However, using them had some challenges. Solid phase microextraction (SPME) techniques are an excellent choice to overcome SPE and LLE restrictions. Sensitivity, eco-friendliness, minimal solvent consumption, and simple operation are among the advantages of these methods. Since Arthur and Pawliszyn introduced SPME in 1990, several studies on various contaminants, including heavy metals, have been done.^15^ In past decades, different sorbents have been developed for SPME extraction and pre-concentration of metal ions. Recent approaches include ionic liquid-based SPME, hollow-fiber SPME, and magnetic dispersive SPME.^16–19^

Polyacrylonitrile (PAN) is a synthetic, semicrystalline organic polymer resin. High mechanical strength, thermal and chemical stability, surface modification capabilities, and fiber-forming capability make it a suitable choice for fiber production in separation and purification processes. PAN is a versatile polymer with exceptional chemical, mechanical, and thermal characteristics as well as a high miscibility with hydrophilic components, which can be produced in large quantities through nitrile polymerization. Because of its inherent toughness and flexibility, it has been widely recognized for producing different fiber and nanofiber structures.

Because of their particular structure and surface area for interaction with solutions, hollow PAN fibers are an ideal choice for environmental purification and pollutant extraction processes as a good sorbent. The extraction effectiveness of pollutants in aqueous media, however, may be impacted by drawbacks such as their surface structure and related hydrophobicity. To improve these issues, doping nanoparticles in PAN bulk solution has been proposed. Nanoparticles can improve the mechanical strength, permeability, selectivity, and electrical conductivity of the PAN matrix.^20^ They can also improve the membrane's antifouling properties and enable better long-term performance by decreasing the tendency of organic species to adhere to the membrane surface. Graphene has drawn great interest in these studies because of its unique electrical, mechanical, thermal, and structural characteristics.^21,22^

Graphene has a large theoretical specific surface area, indicating a high capacity for sorption. Additionally, it may establish a strong π–π stacking contact with the benzene ring because of its large delocalized π-electron system. On the nano-sheet surface of GO, a significant amount of oxygen atoms are present as epoxy, hydroxyl, and carboxyl groups.^20,23^ Pure GO membranes often have low flux levels and limited stability during water treatment, despite these characteristics.^24^

Another suggestion is the modification of the hollow fiber membrane surface. For this purpose, certain materials can be added to the PAN fiber surface. This coating enhances the fiber structure in addition to increasing the surface hydrophilicity. Also, it modifies the selectivity of fiber. Therefore, by improving adsorption capacity and selectivity towards contaminants, these surface changes can increase the potential uses of PAN fibers in water purification and related industries. Few research works have been reported on their surface functionalization.^25–27^

The use of functionalized PAN fiber for the extraction of heavy metals has been rarely presented. In this work, polyacrylonitrile-graphene oxide hollow fibers (PAN/GO HF) were synthesized for extraction and purification applications. Their surfaces were modified by the self-polymerization of tyrosine (Tyr). The surface characteristics of PAN/GO-Tyr hollow fiber membrane were carefully studied. To better understand the adsorption process of heavy metal ions, adsorption isotherms and kinetics were also investigated. Intraparticle diffusion and pseudo-second order models were employed to investigate the adsorption kinetics. Also, the removal and pre-concentration of As^3+^, Sn^2+^, Pb^2+^, and Cu^2+^ metal using PAN/GO-Tyr HF fiber as a SPME sorbent and UV-vis detection procedure were performed. The extraction was performed using 5 mL of aqueous analyte solution at different concentrations (0.001–100 μg L^−1^), contact time (5–20 min), pH (3.5–8.5), and stirring rate of 250 rpm for 40 min. Desorption of the analytes from the adsorbent was done using 0.1 mol L^−1^ HNO_3_ (2–10 mL) solvent at an ambient temperature. The extraction-spectrophotometric procedure identified ultra-trace concentrations of metal ions in water, including As^3+^, Cu^2+^ (1.3 mg L^−1^), Pb^2+^ (0.05 mg L^−1^), and Cu^2+^ (1.3 mg L^−1^), even at levels below the WHO permissible limits. Also, changes in the FTIR spectra and ion removal percentage considerations revealed that the constructed membrane can be used six times for extraction purposes without losing its performance. The findings showed that surface modification significantly improved the hydrophilicity of PAN fibers and increased their adsorption effectiveness, making them a useful choice for water treatment and elimination of different pollutants.

## 2. Materials, experimental and instrumentation

### 2.1 Materials

Polyacrylonitrile (PAN) was purchased from Acryl Company in Isfahan, Iran. SnCl_2_·2H_2_O, CuSO_4_, PbCl_2_, and As_2_O_3_, which were acquired from Merck (Darmstadt, Germany), were dissolved in double-distilled water to prepare stock solutions of metal ions. Aside from other chemicals, Merck (Darmstadt, Germany) provided analytical grade ethanol (EtOH) and dimethylformamide (DMF) with 99.9% purity.

### 2.2 GO synthesis

GO nano-sheets were produced using Hummers' approach. In summary, a mixture of HNO_3_ and H_2_SO_4_ (1 : 3 v/v) was gradually added to 1.0 g of graphite powder, and the mixture was stirred constantly for 24 hours at room temperature. The resulting mixture was heated so that the acid could evaporate. After the remaining powder was dissolved in H_2_SO_4_ in an ice bath, KMnO_4_ (6 g) was added gradually. The resultant mixture was stirred for one hour. After that, 10% v/v hydrogen peroxide solution was added dropwise to the resultant mixture until it took on a bright yellow coloring. After the suspension was continuously rinsed with deionized water until the pH of the precipitates in suspension in pure water was neutral, it was finally dried in a vacuum oven.

### 2.3 PAN/GO hollow fiber synthesis

Firstly, PAN polymer and graphene oxide nanoparticles were dried fully, and moisture was removed by placing them in an oven set to 50 °C for 24 hours. To get a totally homogenous polymer solution, 15 weight percent of PAN polymer and 85 weight percent of DMF were aggressively mixed at 60 °C for three days using a mechanical stirrer. To get rid of any potential air bubbles, the solution was kept at room temperature. Following defoaming, the PAN/GO solution slurry solution was transferred to the spinning tank and continuously swirled for ten hours.^24^

### 2.4 PAN/GO-Tyr HF membrane synthesis

The treatment of PAN/GO involving 10% sulfuric acid was used for the cleaning and activation of PAN/GO HF. Surface activation of the PAN/GO nanocomposite created reactive sites. A 1.0 × 10^−3^ mol L^−1^ solution of tyrosine (Tyr) was prepared and its pH was maintained at 8.5 using 0.1 mol L^−1^ phosphate buffer solution. The PAN/GO HF was immersed in Tyr solution and stirred for 4 h at 30–40 °C. This time allowed the reaction between Tyr and the activated surface to take place, forming covalent bonds or other chemical interactions. Then, modified PAN/GO-Tyr HF was washed several times with deionized water to remove any excess reagents, unreacted Tyr and other impurities. The fabricated fiber was dried for further characterization.

### 2.5 Instrumentation

A model 713 pH per mV meter (Metrohm, Switzerland) was used to control the pH of solutions. A spectrophotometer (Analytik Jena) was used to measure the UV-vis spectra, which were recorded using a quartz cell measuring 1.0 cm, scan rate of 100 nm min^−1^, and slit width of 2.0 mm. The captured spectra were digitized one data point per nanometer. For the FTIR measurement, a Bruker Vector 22 FT-IR with a transmittance mode spectral range from 4100 to 400 cm^−1^ was utilized.

Scanning electron microscopy (SEM) was used to examine the microstructure of the samples (Tescan-Vega, Czech Republic). The micrographs of the modified electrodes were analyzed using scanning electron microscopy (SEM-EDX, XL30, Philips Netherlands). To capture an electron microscope picture, an accelerating voltage of 80 kV was applied to a Zeiss-EM10C.

### 2.6 Extraction procedure

The hollow fibers were manually cut into 2 cm pieces, cleaned of contaminants with acetone, and then allowed to air dry. The acceptor phase, 6.0 μL of EtOH (Merck) solution, was introduced into the hollow fiber's lumen. To stop leaks, the fiber's two exposed ends were sealed. The produced fiber was put in a glass vial and exposed to 5 mL of an aqueous analyte solution at concentrations between 0.001 and 100 g L^−1^, contact times between 5 and 20 minutes, pH between 3.5 and 8.5, and a desorption solvent volume between 2 and 10 mL for 0.1 mol L^−1^ HNO_3_ at room temperature.

After covering the vial, the solution was stirred for 20 minutes at 250 rpm. The analytes diffused through the porous hollow fiber from the solution to the sorbent during the proper time extraction. After that, the fiber was removed from the vial, placed in a new vial, and cleaned using a 3 mL HNO_3_ solution to aid in the analytes' desorption. Ultimately, the solution was mixed with a molar ratio of tyrosine reagent, and the absorbance of the complexes was measured between 200 and 800 nm. All of the aforementioned components, aside from the analytes, were used to run the blank solutions for each analysis. The UV-vis spectra of metal–tyrosine complexes are given in Fig. S2.†

### 2.7 Kinetic experiment measurements

Adsorption kinetic experiments were applied to determine the adsorption rate for PAN/GO-Tyr HF towards metal ions. For each metal ion, 2 cm PAN/GO-Tyr HF was immersed in a 10 mL solution containing 5.0 × 10^−5^ mol L^−1^ of ion at pH 6.8. The solution was shaken at 250 rpm speed under room temperature and desorption was performed using HNO_3_ solution. Then, the absorbance of metal ions after complexation with a stoichiometric amount of Tyr was recorded at certain interval times until the saturated adsorption was acquired. The kinetic study determined the biosorption type (chemisorption or adsorption). The amounts of adsorbed ions were determined using the following equations.1
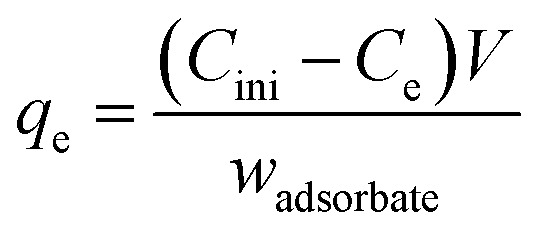


The following flowing equation was used to get the sorption value of the metal ion at time *t*, or *q*_t_.2
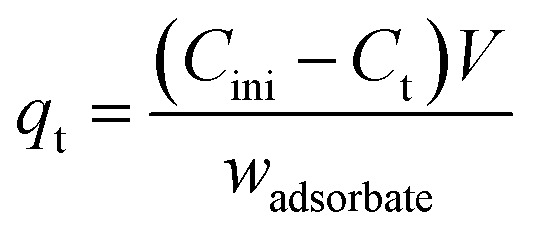


The concentration of metal ions at any given moment is *C*_t_ (mg L^−1^).

The removal was calculated using the following equation.3
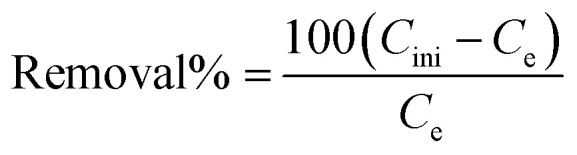


## 3. Results and discussion

### 3.1 Structural characterization of the sorbent

#### 3.1.1 FTIR characterization

**Fig. 1:**
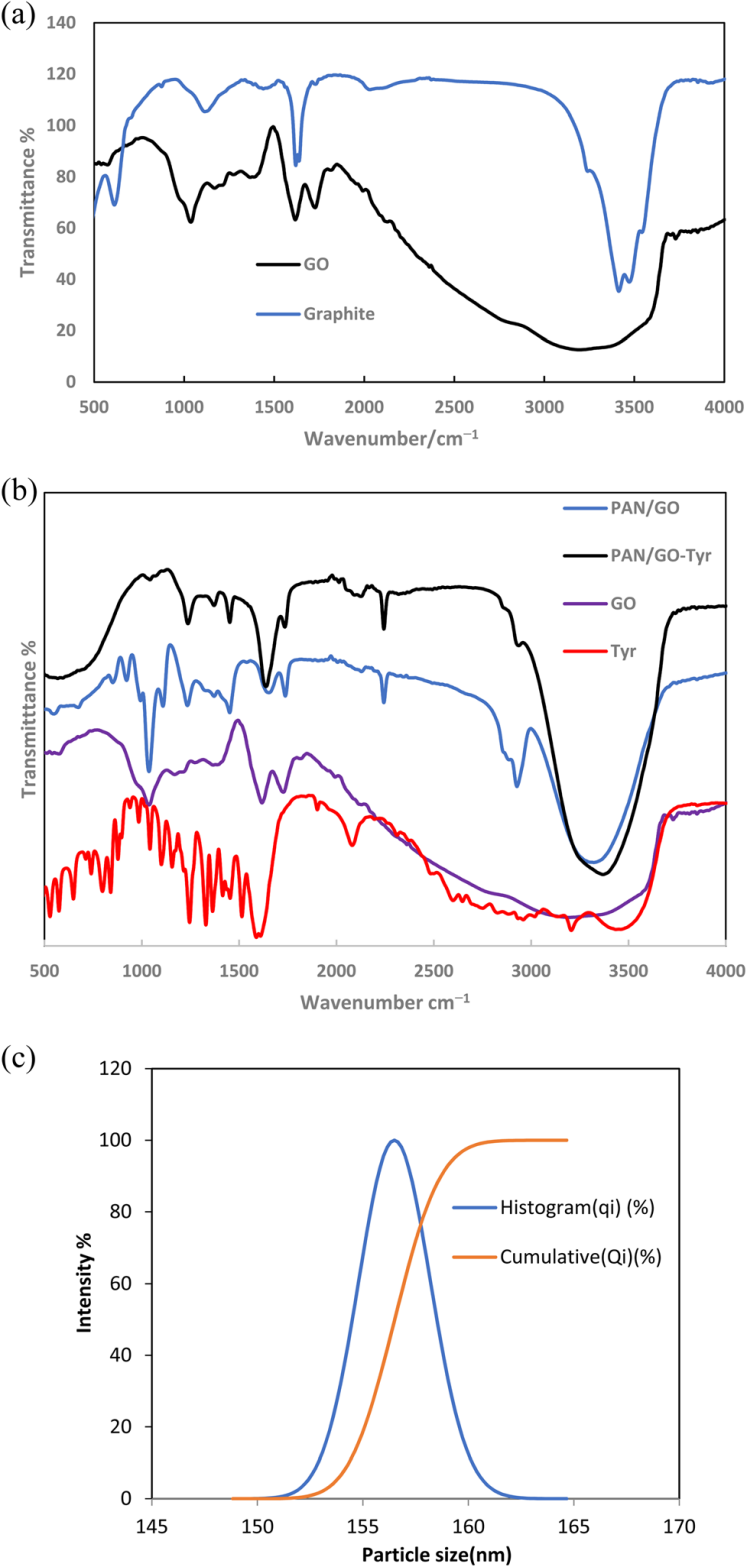
FTIR spectra of (a) pure graphite (blue line) and synthesized GO (black line); (b) FTIR spectra of PAN/GO, tyrosine and PAN/GO-Tyr HF and (c) DLS image of GO nanoparticles.

The FTIR spectrum of graphite is shown in Fig. 1a. According to this figure, the peak is often associated with the stretching vibration of C–C bonds in graphite, observed at 1091 cm^−1^. Also, the peak exhibited at 1409 cm^−1^ is attributed to the vibration of the graphite lattice structure, particularly the stretching vibration of the carbon–carbon bonds in the hexagonal rings. The FTIR spectra of synthesized GO was considered. The vibrational peaks of epoxy (C–O) groups, alkoxy (C–O) groups, and carboxyl (C=O) groups are exhibited at 1039 cm^−1^, 1166 cm^−1^ and 1367 cm^−1^, respectively. The distinctive stretching vibration peaks of the aromatic ring's C=C bond (sp^2^ carbone skeleton) is located at 2026 and 1621 cm^−1^. The carbonyl (C=O) groups' vibrational peak was measured at 1729 cm^−1^. The broad exhibits peaks from 3040 to 3380 cm^−1^ in the high frequency range due to the stretching mode of the O–H bond.^22,28^

The FTIR spectrum of PAN/GO is given in Fig. 1b (dark blue spectrum). The peaks at about 921 cm^−1^ and 1238 cm^−1^ are associated with C–N bending and C–N stretching, respectively, while 2902 and 2929 cm^−1^ could be associated with C–H stretching. These peaks are typical of PAN. Additionally, GO is characterized by peaks at about 1035 cm^−1^ (C–O stretching), 1369 cm^−1^ (C–H bending in aromatic ring), 1664 cm^−1^ (C=C stretching in aromatic ring), 1741 cm^−1^ (C=O stretching), and 2244 cm^−1^ (C≡N stretching). The peaks at 993, 1110, 1452, 2902, and 854 cm^−1^, as well as between 3260 and 3380 cm^−1^, may be related to both PAN and GO, or they may show overlapping bands. Therefore, the existence of peaks at about 1035, 1369, 1664, 1741, and 2244 cm^−1^ indicates the particular presence of GO in the PAN structure of PAN/GO hollow fiber. The shifting of peaks in the FTIR spectra of PAN/GO nanocomposite can provide valuable information about changes in the molecular structure or interactions between molecules. The absorption peak at 1050 cm^−1^ increased gradually after the incorporation of GO. This suggests a shift towards higher wavenumbers, indicating a change in the bonding environment, possibly due to interactions involving the carbon–oxygen bonds in GO's C–O–H structure. The absorption peak at 1738 cm^−1^ also increased gradually, indicating a shift towards higher wavenumbers, suggesting changes in the carbon–oxygen double bond in GO. The absorption wide peak at 3419 cm^−1^ increased gradually and shifted towards higher wavenumbers, indicating changes related to the absorption characteristic peak of hydroxyl groups.

Fig. 1b (red spectrum) reveals the FT-IR spectrum of tyrosine. The peaks at 574 cm^−1^ and 647 cm^−1^ are associated with out-of-plane bending of the aromatic ring. The vibrational peaks at about 840 cm^−1^ and 877 cm^−1^ may correspond to C–H out-of-plane vibrations in the benzene ring 1041 and 1051 cm^−1^ can be attributed to C–O stretching in the phenolic group. Also, the peak at about 1155 cm^−1^ is due to C–H in-plane bending or C–O stretching and 1243 cm^−1^ usually corresponds to phenolic C–O stretching. The peak at 1328 cm^−1^ may be due to C–N stretching or O–H bending. Aromatic ring stretching vibrations appeared at 1508 and 1513 cm^−1^. Also, the N–H bending in amine groups or C=C stretching in the aromatic ring was exhibited at 1558 cm^−1^. The vibrational peaks at 1903 cm^−1^, 2078.8 cm^−1^ and 2497.4 cm^−1^ can be due to an overtone or combination band. The C–N stretching or aromatic C–H in-plane bending vibrational peak was measured at 1101 cm^−1^.

In the FTIR spectrum, the broad peak between 2500 and 3600 cm^−1^ is due to the overlap of the O–H stretching peak of –COOH, N–H stretching and the phenolic O–H groups.

The main wavenumbers of PAN/GO-Tyr HF in the FTIR spectrum (Fig. 1b black spectrum) are given in Table 1. The peak at 1236 cm^−1^ is typically associated with the stretching vibration of the C–N bond, which could also indicate the presence of amine (NH_2_) groups or possibly amide groups. The broad peak at about 1640 cm^−1^ suggests the presence of amide bonds, characteristic of proteins, indicating the formation of amide bonds before attachment to the PAN/GO surface. The broadening of this peak could indicate hydrogen bonding. Also, the wavenumber 1735 cm^−1^ is associated with the stretching vibration of the carbonyl (C=O) group, which further supports the presence of amide bonds. Hence, the results suggest that tyrosine was attached to the surface from its carboxylic group based on the presence of the amide bonds, indicated by the broad peak at about 1640 cm^−1^ in the FTIR spectrum. This peak suggests the formation of amide bonds, which typically occurs between a carboxylic acid (COOH) group and an amine (NH_2_) group. In the case of tyrosine, the carboxylic acid group (–COOH) could react with another functional group on the surface, such as an amine group, to form an amide bond (–CONH–), attaching tyrosine to the surface. Based on these peaks, it seems likely that amide bonds are present on the surface, indicating the formation of amide bonds before attachment to the PAN/GO surface. This is supported by the broad peak at about 1640 cm^−1^ and the presence of other peaks characteristic of amide bonds. Additionally, other functional groups such as carbonyl (C=O), nitrile (C≡N), and aliphatic C–H bonds are also present on the surface.

**Table 1:** Main functional groups present on PAN/GO-Tyr hollow fiber and their peaks and wavenumbers

Wavenumber (cm^−1^)	Functional group	Wavenumber (cm^−1^)	Functional group
1039	C–N stretching	1640	Amide (C=O–NH) bonds
1236	Amide (C=O–NH) group	1735	Carbonyl (C=O) group
1374	Bending vibration of C–H or C–N bond	2242	Stretching vibration of nitrile (C≡N) group
1454	C–H bond of aromatic ring	2857 and 2940	C–H bond of aliphatic group

#### 3.1.2 Dynamic light scattering (DLS)

The powerful dynamic light scattering (DLS) technique was used to investigate the size of the synthesized GO nanoparticles. The results of this method can help to determine the uniformity and size dispersion of graphene oxide sheets. The DLS curve shows the synthesized GO nanoparticles. A symmetrical distribution can be seen in the DLS curve, where the particles are uniformly distributed around an average value of 156 nm (Fig. 1c). These sizes are slightly larger than the actual size of the graphene oxide sheets, which is due to the surface effects and water layers around the GO particles in the solution.^24^

#### 3.1.3 XRD analysis

**Fig. 2:**
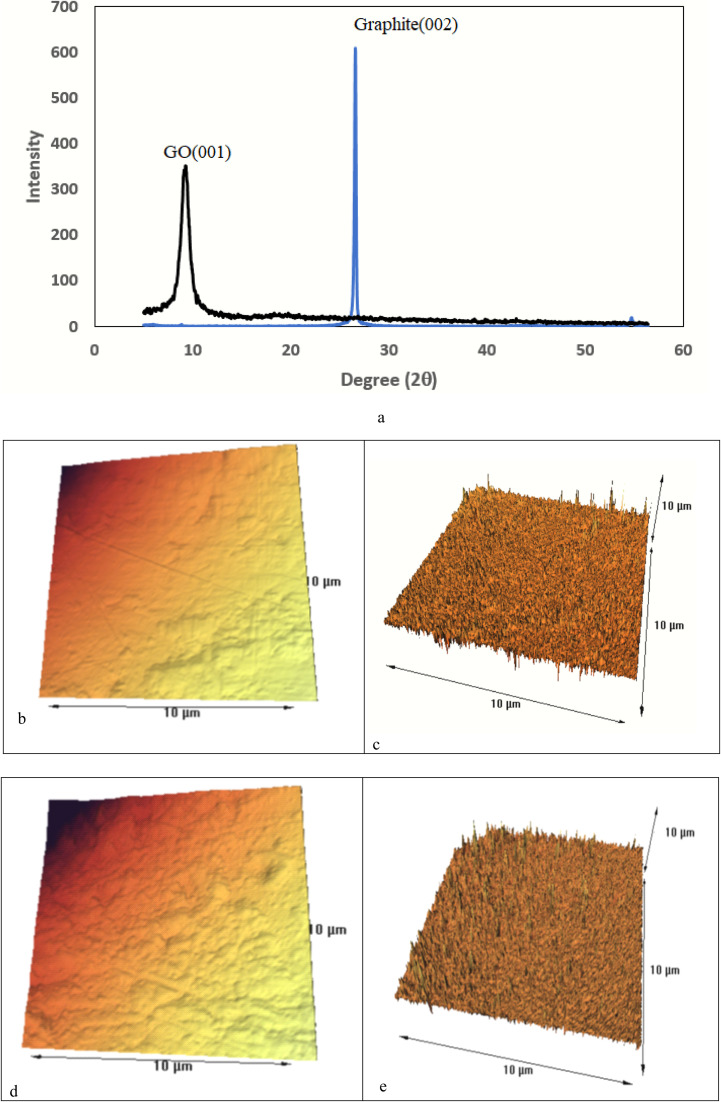
(a) XRD spectra of pure graphite (black line) and GO (blue line), (b) AFFM 2D image and (c) 3D image of PAN/GO HF and (d) 2D AFM image and (e) 3D image of PAN/GO-Tyr HF.

The XRD spectra of graphite powder and synthesized GO are presented in Fig. 2. The peak at 2*θ* = 26.6° proves the presence of ordered layers in the graphite structure. This peak corresponds to the (002) plane reflection and is a main property of the layered structure of graphite. Since carbon atoms are ordered in overlapping layers and weak forces bond the layers together, this plane shows the distance between these layers. The peak at 2*θ* = 9.3°, belongs to the (001) plane reflection and shows a different structure of GO with respect to pure graphite. Since, GO includes different functional groups, *e.g.*, hydroxyl and epoxide groups, on the basic plane and its edges. This peak is probably related to the interlayer spacing in the GO structure and due to the presence of oxygen-containing functional groups is usually larger than that of pure graphite. As a result, the difference between peak positions in graphite and GO XRD spectra depicts the structural differences between them. Graphite with a layered structure shows a sharp characteristic peak, while GO presents a broader peak due to the presence of oxygen functional groups.

#### 3.1.4 AFM characterization

**Fig. 3:**
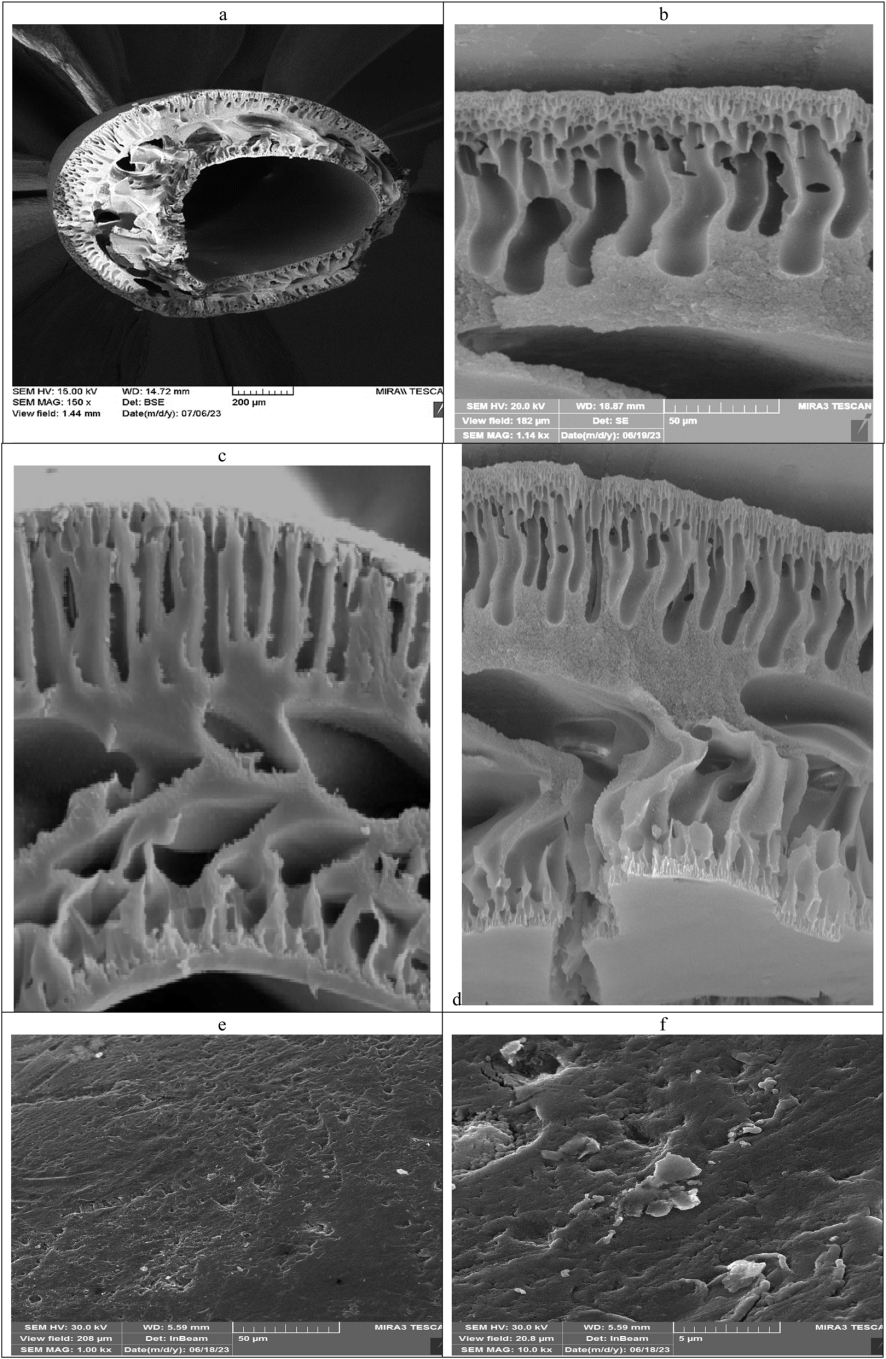
FESEM image of PAN/GO-Tyr (a), cross-sectional images (b) and (d), cross section of PAN/GO hollow fiber (c) and surface images of PAN/GO-Tyr HF (e) and (f).

An important parameter that describes the surface irregularity at the microscopic level is the surface roughness. The higher the roughness value, the more irregular the surface, while a low roughness value indicates a smooth surface. The AFM images of PAN/GO HF and PAN/GO-Tyr HF are shown in Fig. 3. Average roughness values are 12.8 for PAN/GO and 4.8 for PAN/GO-Tyr HF. These values show that the surface roughness is reduced in the presence of tyrosine. The PAN/GO HF roughness value means that the target surface has an irregular structure or texture unlike PAN/GO-Tyr HF. This reduction indicates that tyrosine has been able to form a layer(s) on the PAN/GO surface, which acts as a protective surface and reduces the surface roughness. Tyrosine can chemically interact with the surface of the hollow fiber and bonding it through hydrogen bonds. Therefore, its presence as a surface coating or modifying agent has potentially altered AFM measurements. Examining the FTIR results showed that chemical changes occurred on the PAN/GO HF surface and amide bonds were formed in the presence of tyrosine. The peaks related to amide and carbonyl functional groups confirmed this idea. Now, by comparing the findings of AFM with FTIR results, it was concluded that the modified surface shows a uniform surface distribution and reduction in surface roughness with chemical changes and functional groups. Therefore, tyrosine and the formation of amide bonds have made the surface of the fiber more homogeneous and the morphology of the surface smoother.

#### 3.1.5 FESEM analysis

FESEM was used to examine the morphologies of PAN/GO-Tyr HF and PAN/GO HF membranes. The cross section and surface images are shown in Fig. 3. This figure shows that the PAN/GO-Tyr HF membrane had a four-layer structure through their respective cross sections view. It can be clearly seen from the enlarged pictures of the cross sections (Fig. 3c) that the outer layer with very small pores was formed in the presence of tyrosine. Since PAN/GO HF had three layers including middle finger-like layer, which is sandwiched between the inside skin layer and outside skin layer of hollow fiber (Fig. 3d). According to the figures, a significant change, thumb-like pores, is visible in the outside layer of the membrane.

#### 3.1.6 BET analysis

**Fig. 4:**
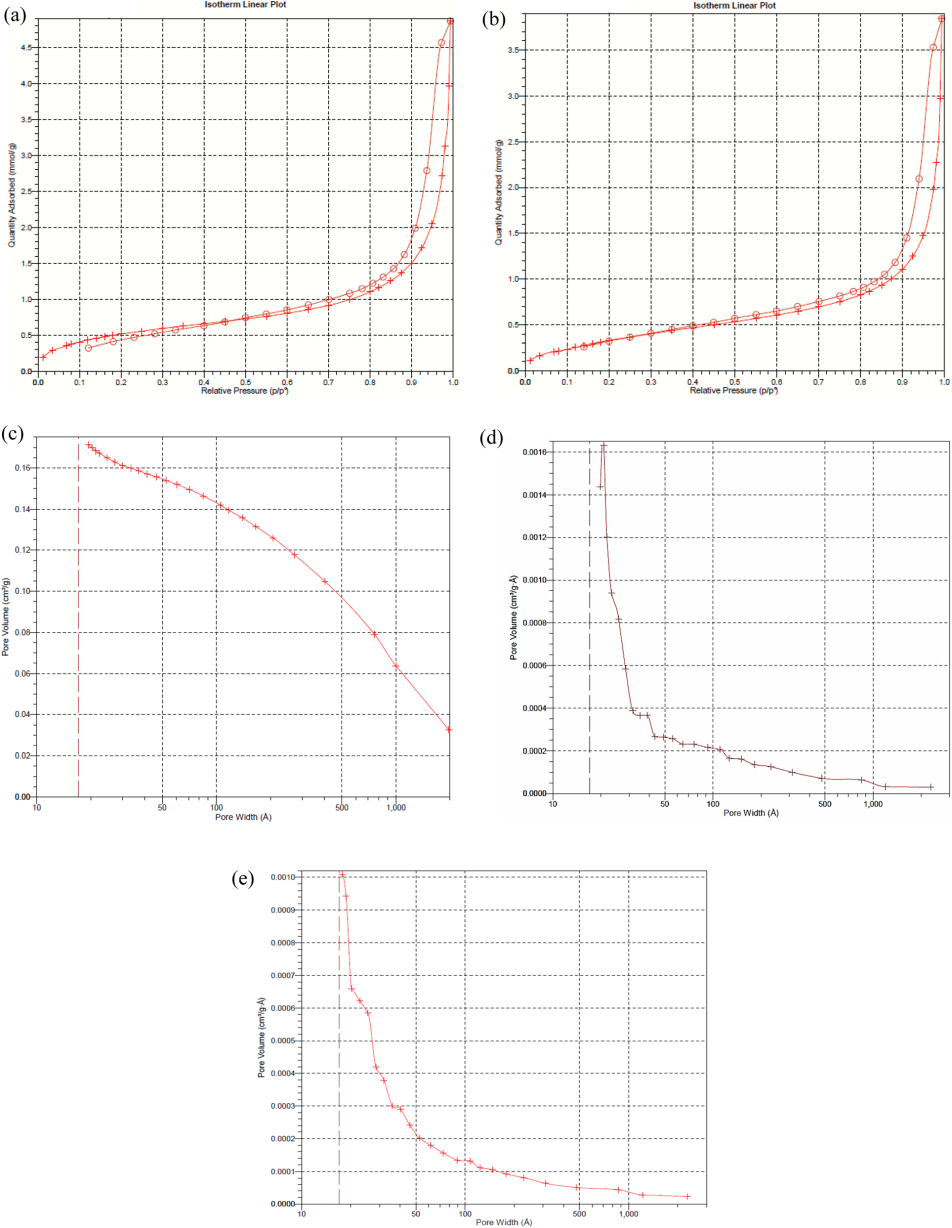
BET N_2_ adsorption–desorption isotherms of PAN/GO-Tyr (a) and PAN/GO HF (b), BJH adsorption cumulative pore volume of PAN/GO-Tyr (c), BJH adsorption d*V*/d*w* pore volume of PAN/GO-Tyr (d) and BJH adsorption d*V*/d*w* pore volume of PAN/GO HF (e).

Investigating the surface characteristics and pore structure of porous materials can be facilitated using the nitrogen adsorption–desorption isotherm diagram. This graph illustrates how relative pressure affects the amount of nitrogen absorbed. Numerous details about the size, volume, and specific surface area of pores, as well as their kind can potentially be taken in this diagram. Fig. 4 shows the nitrogen adsorption–desorption isotherm diagram of PAN/GO-Tyr HF (Fig. 4a) and APN/GO HF (Fig. 4b). Based on the shape and characteristics of this graph, it can be said that in both cases, the isotherms are probably of type IV, which indicates mesoporous materials with pores between 2 and 50 nm.^29^ The presence of a hysteresis loop in this type of isotherm indicates a complex pore structure, such as interconnected pores or narrow bottlenecks, which causes the desorption process to be delayed. This isotherm behavior is common for mesoporous materials. However, there are differences in the adsorbed quantities and the shape of the hysteresis loop. A more prominent hysteresis loop can be seen in the PAN/GO-Tyr HF diagram, while in PAN/GO HF, the hysteresis loop is smaller. This shows that the PAN/GO-Tyr HF membrane has more complicated pores and the pores have changed in such a way that they have increased the hysteresis. Also, both isotherms show adsorption at low relative pressures, but the PAN/GO-Tyr HF graph has a slightly higher absorbance, indicating the possibility of a larger number of finer pores or a change in the sample surface. It is in agreement with the results of the FESEM morphology investigation (Fig. 3c), which shows the existence of a large number of small pores in the second layer formed in the presence of tyrosine. BET analysis results are shown in the Table 2. According to the table, the increase in the specific surface area in the modified fiber in the presence of tyrosine grafting is usually the result of the creation of new, porous and nanometer structures on the surface, which led to an increase in surface accessibility for gas adsorption. The BJH adsorption cumulative pore volume curve of PAN/GO-Tyr HF is shown in Fig. 4c. The structural properties of the membranes, such as the distribution of pore sizes and specific surface area, may be illustrated with this diagram. According to Fig. 4c, the curve has a steep slope at sizes below 20 Å that rises rapidly, which indicates that most of the cumulative volume of pores is located in micropore sizes. Therefore, this membrane is predominantly microporous and has a high contact surface for the adsorption of molecules. The derivative d*V*/d*w* pre volume of BJH adsorption of PAN/GO-Tyr shows that there is a variety of pore diameters, with the size of the pores ranging from about 10 to over 100 Å (Fig. 4d). However, a microporous structure is indicated by a prominent peak at small widths (about 10–20 Å). This signifies that the majority of the pores in this membrane are small and provide a high specific surface area. It shows that there is a smaller number of larger pores in the PAN/GO-Tyr HF. Also, it is clear that for widths above 1000 Å, the volume of pores in this range is very small. The derivative curve for PAN/GO fiber also shows a sharp peak in the width of small pores (about 10 Å), indicating a high volume of small pores in this membrane (Fig. 4e). Also, in the PAN/GO derivative curve, more peak dispersion and lower intensity is observed, which indicates that this material has both micropores and mesopores, although micropores are dominant.

BET analysis results for PAN/GO and PAN/GO-Tyr hollow fibers

**Table:** 

Surface area (m^2^ g^−1^)					
Hollow fiber	BJH adsorption cumulative surface area of pores	BJH desorption cumulative surface area of pores	t-Plot external surface area	BET	Langmuir
PAN/GO/Tyr	43.12	47.77	52.91	44.91	65.54
PAN/GO	33.81	36.09	45.44	30.71	47.37

**Table:** 

Pore volume (cm^3^ g^−1^)		
Hollow fiber	BJH adsorption average pore volume	BJH desorption average pore volume
PAN/GO/Tyr	0.172	0.166
PAN/GO	0.133	0.132

**Table:** 

Average pore size (Å)			
Hollow fiber	BJH adsorption average pore width	BJH desorption average pore width	BET, average pore width
PAN/GO/Tyr	158.78	139.17	83.83
PAN/GO	158.03	145.93	89.29

### 3.2 Extraction of metal ions by PAN/GO-Tyr HF

At the beginning of the extraction and removal testing, metal ions were studied using PAN/GO hollow fiber. Extraction was performed using the mentioned protocol and desorption was performed. The UV-vis spectra of desorbed ion complexes were recorded. The absorbance spectra are shown in Fig. S1.† The results showed that the removal of metal ions is less than 20%. Therefore, modification of the surface of the manufactured hollow fiber became the priority of our research study.

Surface characterization reveals that the fabricated nanocomposite adsorbent includes oxygen-containing functional groups, such as carboxyl, epoxy, and hydroxyl. Hence, the adsorption ability of metal ions by PAN/GO-Tyr HF by functionalizing with GO, a two-dimensional material with a large surface area and incorporating certain functional groups of tyrosine, could be increased. Therefore, the preconcentration and removal of As^3+^, Cu^2+^, Pb^2+^ and Sn^2+^ metal ions were examined. In the following, we will explain the extraction process and pre-concentration of these metals. Also, the adsorption mechanism will be introduced.

#### 3.2.1 Effect of experimental conditions

It has been reported that experimental factors such as pH of the feed solution, extraction solvent, feed solution volume, extraction time and stirring rate affect the extraction efficiency. Hence, these experimental parameters that play a crucial role in the performance of the PAN/GO-Tyr HF adsorbent for the extraction of selected metal ions, were carefully studied in order to achieve the maximum extraction efficiency of analytes from the water sample matrices. The optimization procedure has been performed using the one factor at a time approach.

##### 3.2.2 Primary consideration of solvent in the PAN/GO/Tyr HF lumen

**Fig. 5:**
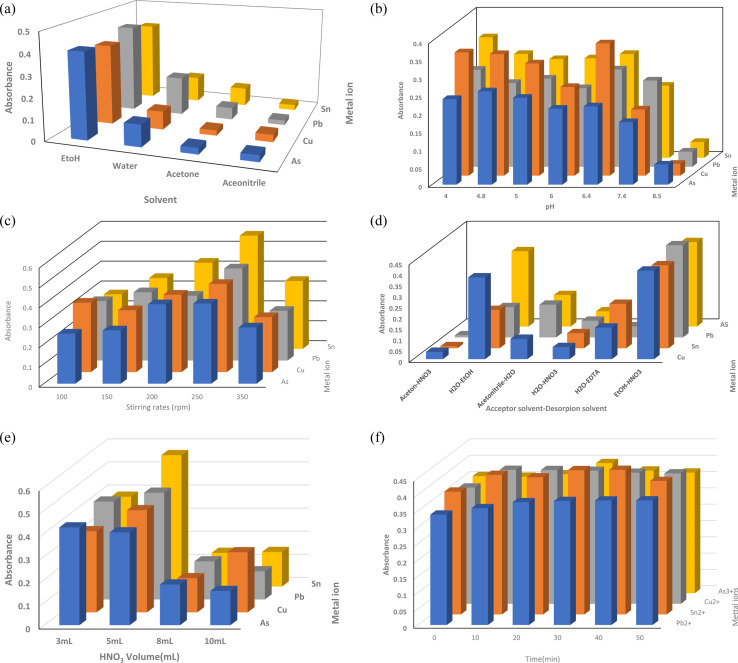
(a) Effect of organic solvent in the lumen, (b) effect of feed solution, (c) effect of sample agitation, (d) effect of the desorption phase, (e) effect of desorption solvent volume and (f) effect of contact time on the extraction of metal ions using PAN/GO-Tyr HF adsorbent.

A compromise may need to be made occasionally, especially when extracting a group of compounds with varied polarity since some may have a stronger extraction affinity for a different solvent. Organic solvents with high extraction efficiency (enrichment) for the metal ions should be utilized. A suitable organic solvent should be immiscible with the donor solutions, have a high boiling point to minimize volatile and diffusion losses during extraction, and be compatible with the fiber in order to maximize its efficiency in extracting analytes of interest.^30^ Due to the presence of PAN, there was also a limitation in the selection of some solvents. Hence, the type of acceptor solvent in the HF lumen was studied. According to the results of Fig. 5a, ethanol is suitable for the extraction of metal ions using fabricated HF.

Solvents such as ethanol help metal ions interact with hollow fiber adsorbents. In the extraction procedure, it releases adsorbed metal ions and improves the efficiency and selectivity of the extraction. Ethanol can have an impact on the kinetics of the extraction procedure as well as the rate of metal ion adsorption and desorption. Additionally, by acting as a stabilizing solvent, ethanol aids in the extraction of metal ions by limiting precipitation and unfavorable reactions.

##### 3.2.2.1 Selection of pH

Sample pH is an important factor that affects the extraction recovery of analytes and can also improve the sensitivity of measurements. Therefore, its effect was investigated as the first factor. The impact of pH of the donor phase on the absorbance of metal ions is seen in Fig. 5b. By adding proper amounts of sodium dihydrogen phosphate and phosphoric acid or acetic acid and sodium acetate, the pH was measured in the range of 4.0–8.5. Solutions containing sodium hydroxide or hydrochloric acid were employed to modify pH. It is clear that under other circumstances, the absorbance of Cu^2+^, Pb^2+^, and Sn^2+^ ions was greater when the pH of the donor phase was 6.2.

For As^3+^, although the absorbance value of pH 4.8 is slightly higher than that of pH 6.2, it does not have a significant difference. Therefore, pH 6.8 was used as the optimum pH for the subsequent investigation of the extraction process. Under neutral pH conditions, PAN does not significantly contribute to surface charge. Graphene oxide and tyrosine have partially deprotonated oxygen functional groups, resulting in a negatively charged surface. At pH 6.8, both groups are predominantly in their deprotonated forms. The overall charge on the PAN/GO-incorporated tyrosine nanocomposite surface at pH 6.8 would be negative, depending on the relative proportions of GO and tyrosine in the nanocomposite and their respective ionization states. Thus, by increasing adsorption sites, the amounts of metal ions adsorbed onto the PAN/GO-Tyr HF adsorbent will significantly increase. Small amounts of metal ions may be adsorbed onto the fibers when the initial pH is less than 4.0 due to competitive adsorption between the available H^+^ ions and metal cations.^25^ The extraction effectiveness may be reduced by the strongly basic pH causing insoluble metal hydroxides to develop in the solution. Hence, pH values higher than 8.5 was not examined.

##### 3.2.2.2 Sample agitation effects

A range of stirring speeds (100–350 rpm) was assessed. As might be predicted, agitating the sample can expedite the kinetics of extraction, which could significantly increase the extraction efficiency. As a result of air bubbles forming and pre-concentration factors decreasing at higher stirring speeds, it was shown that the extraction efficiency improved with stirring rate up to 250 rpm and subsequently dropped with further rate increases. These data led to the selection of 250 rpm as the stirring rate for all the ensuing trials (Fig. 5c).

##### 3.2.2.3 Effect of the desorption phase and its volume

Additionally, the impact of desorption solvent volume on metal ion extraction efficiency was investigated (Fig. 5d). The portion of hollow fiber remained constant in length; therefore, the lumen phase volume remained constant at 6.0 μL. Using HNO_3_ as a desorption solvent allowed for the steady expansion of enrichment factors. The HNO_3_ volume was raised to 10 mL from 3 mL. At 5 mL, the enrichment factor reached its maximum. More than 5.0 mL of sample volume, however, results in poor analyte absorption. The PAN/GO-Tyr HF capacity saturation for a large sample volume might be the cause of this phenomena. Thus, 5.0 mL was chosen as the ideal desorption solvent volume (Fig. 5e).

##### 3.2.2.4 Effects of contact time

The extraction of heavy metals utilizing PAN/GO-Tyr hollow fibers is certainly affected by the contact or adsorption time. Using a variety of interactions, including physical adsorption, chemical adsorption, complexation, and ion exchange, the heavy metal ions in the solution attach to the surface of the functionalized hollow fibers during the surface adsorption process of the metals under study. While physical adsorption often relies on non-specific forces and is weaker, chemical adsorption is specific and stronger since it creates new chemical structures by bonding the adsorbate and adsorbent together. The speed of this adsorption depends on several factors such as the concentration of metal ions in the solution, surface and porosity of the hollow fibers, and most importantly, contact time or adsorption time. Initially, when the functionalized hollow fibers are introduced into the metal ion solution, adsorption takes place rapidly due to the availability of active sites on the hollow fiber surface. However, as time passes and the concentration of metal ions in the solution decreases to a level where the adsorbed metal ions on the fiber surface approach equilibrium, the rate of adsorption may slow down. Hence, longer contact times do not necessarily lead to better extraction efficiency beyond a certain point, as the system may reach equilibrium and further adsorption becomes negligible. Conversely, shorter contact times may result in incomplete extraction and leave significant amounts of heavy metals in solution. In conclusion, contact time or adsorption time significantly affects the extraction efficiency of heavy metals using PAN/GO-Tyr HF. Hence, contact time as an important factor affecting the efficiency of adsorption and was carefully optimized.

According to Fig. 5f, the absorbance increases with an increase in contact time up to 15 min for As^3+^, Cu^2+^ and Sn^2+^ ions and 25 min for Pb^+2^, after which it is more or less constant. The results, indicated that the absorbance values increased by increasing the extraction time. The higher adsorption at the initial contact time could be related to the influence of mass transfer driving force of heavy metal ions into the surfaces of Tyr and the abundance of active sites on PAN/GO-Tyr HF and the adsorbent.^31^

Because there was less mass transfer acting as a driving factor, the absorbance started to stabilize or decline after 30 minutes of extended extraction time. In the end, 25 minutes was decided upon as the extraction time for additional examinations.

### 3.3 Method validation

Under optimal experimental settings, the suggested method's analytical performance was validated. The calibration graphs were created by extracting analytes at varying concentrations from aqueous solutions. Table 3 displays the linear dynamic ranges, graphs' linearity, and limits of detection (LODs). Also, the enrichment factor of analysis was calculated. According to the calculation, the EF values were 240.6, 308.5, 545.8 and 322.9 for As^3+^, Cu^2+^, Pb^2+^ and Sn^2+^ ions under the optimum conditions, respectively.

**Table 3:** Figure of merits of the proposed method for the pre-concentration of As^3+^, Cu^2+^, Sn^2+^ and Pb^2+^ using PAN/GO-Tyr HF

Metal ion	Linear range (ppb)	Equation	*r* ^2^	Detection limit (ppb)
As^3+^	0.005–4.0	*A*_As_ = 21.4 × 10^3^*C*_As_ + 0.333	0.989	0.004
Cu^2+^	0.01–10	*A*_cu_ = 11.7 × 10^3^*C*_Cu_ + 0.334	0.974	0.005
Sn^2+^	0.003–20	*A*_Sn_ = 2.0 × 10^3^*C*_Sn_ + 0.315	0.999	0.002
Pb^2+^	0.05–1.0	*A*_Pb_ = 11.3 × 10^4^*C*_Pb_ + 0.314	0.989	0.01

The performance of the PAN/GO-Tyr HF-UV-vis detection method was compared with that reported previously. Good EF values were obtained for the PAN/GO-Tyr HF sorbent in comparison with the prevalent sorbents. Table 4 illustrates that the developed method's LOD is lower than the reported methods' LOD, most likely as a result of the developed method's high EF.

**Table 4:** Comparison of the proposed method with some previously reported methods

Adsorbent	Detection method	Removed ions	Linear dynamic range (ppb)	Enrichment factor	LOD (ppb)	Reference
Amine-functionalized TiO_2_ NPs	Colorimetric assay	Cu^2+^	—	—	0.5	32
		Hg^2+^				
Fe_3_O_4_@SiO_2_-ChPr-l-Cys	FAAS	Hg^2+^	0.0050–0.02	—	0.004	33
		Cu^2+^	0.50–100		0.091	
		Mn^2+^	0.50–100		0.221	
		Zn^2+^	0.50–100		0.145	
		Pb^2+^	0.50–100		0.260	
		Fe^2+^	0.50–200		0.0.2532	
GO-SH sorbent	ICP-OES^a^	Hg^2+^	1–1000	—	0.2	34
		As^3+^	1–1000		0.2	
Fe_3_O_4_@SiO_2_/GO-NH_2_	Graphite furnace atomic absorption	As^3+^	1.02 nM	392	1.02 ng L^−1^	35
PAN/GO-Tyr HF	Spectrophotometric	As^3+^	0.005–4.0	240.6	0.004	Current work
		Cu^2+^	0.01–10	545.8	0.005	
		Sn^2+^	0.003–20	322.9	0.002	
		Pb^2+^	0.05–1.0	308.2	0.01	

a Inductively coupled plasma optical emission spectrometer.

### 3.4 Selectivity

**Fig. 6:**
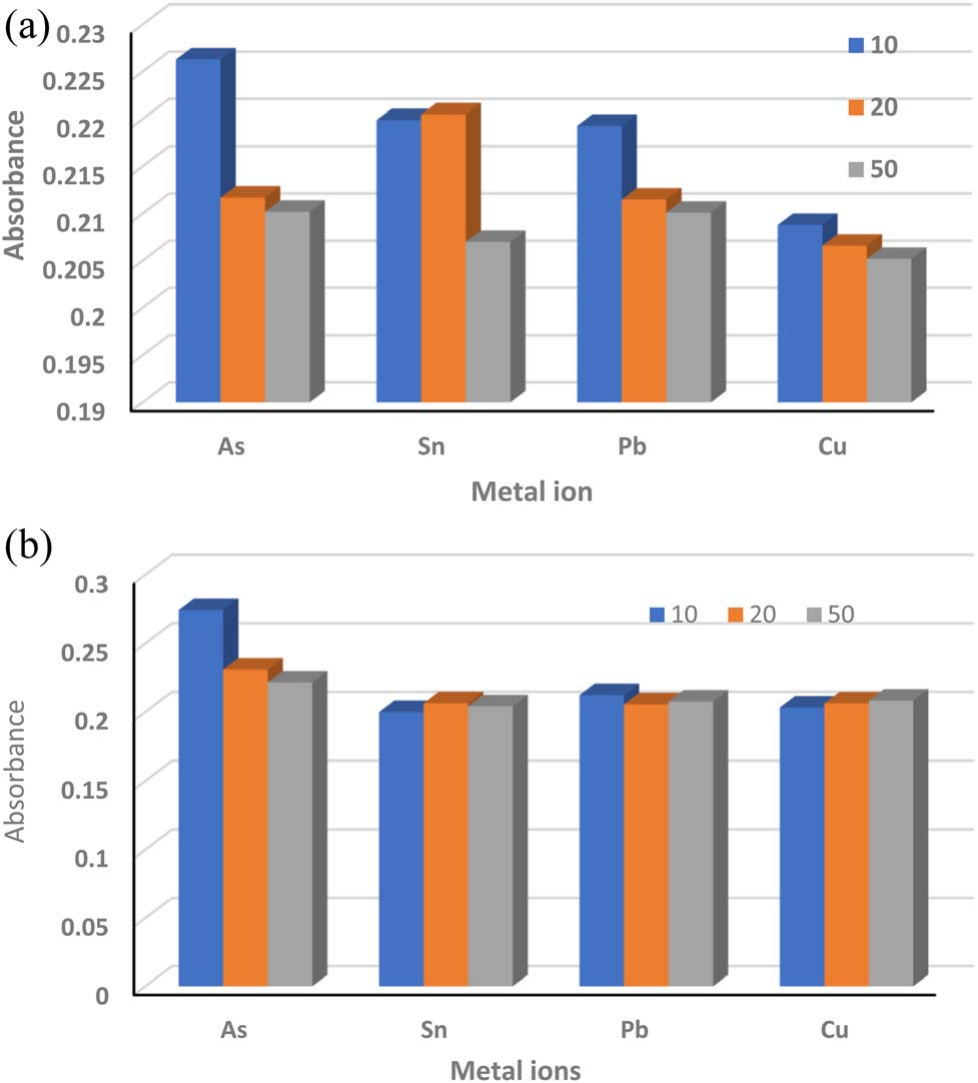
Effect of phosphate ion (a) and carbonate ion (b) on the extraction of metal ions using PAN/GO-Tyr HF.

Phosphate and carbonate anions, may either compete with or interfere with the adsorption of metal ions as they are generally found in an aqueous medium. Thus, their effect on adsorption was investigated at different ratios of anions at optimum conditions. The results are shown in Fig. 6. The decrease in absorbance of the solution could be attributed to these factors: (I) competition of phosphate and carbonate ions with metal ions for adsorption on the adsorbent sites and (II) complex formation between metal ions and anions which the complexes show different absorption characteristics with respect to free metal ions.

### 3.5 Stability of PAN/GO-TYR HF

Experiments on long-term stability and reusability were performed to understand the capacity of PAN/GO-Tyr HF as an adsorbent for SPME method. Also, the FTIR spectrum of used PAN/GO-Tyr was measured and compared with that of the unused adsorbent. The FTIR spectrum is shown in Fig. S3.† According to this figure, some peaks was shifted and intensities was changed. The observed shifts and changes in intensity in the FTIR spectrum of PAN/GO-Tyr HF after 6 cycles compared to unused PAN/GO-Tyr HF suggest variations in the chemical structure and interactions within the structure. The FTIR spectrum of PAN/GO-Tyr HF shows shifts and changes in intensity after six cycles, suggesting structural modifications and chemical interactions within the material. The increase in peaks at 1640 cm^−1^ could indicate an increase in the concentration or structural rearrangement of functional groups, possibly related to stretching vibrations of carbonyl groups or aromatic C=C bonds in tyrosine or PAN/GO moieties. The decrease in peaks at 2240 cm^−1^ suggests a reduction in the concentration or alteration of the C–N bond, possibly due to chemical reactions. Also, the removal percentage of metal ions after 6 cycles is shown in Fig. S4.†

### 3.6 Proposed mechanism

When PAN/GO HF are modified using tyrosine and phosphate at 60 °C, amide bonds or other chemical bonds are likely to form between the carboxyl amino acid groups and functional groups on the GO or PAN surface. Therefore, there is an expectation of significant surface changes in the hollow fiber structure, which were well-illustrated by the results of the modified fiber structure's advanced methods of surface investigation. Carboxyl groups on tyrosine can react with hydroxyl groups on GO surface. This reaction can lead to the formation of ester or amide bonds.

**Scheme 1:**
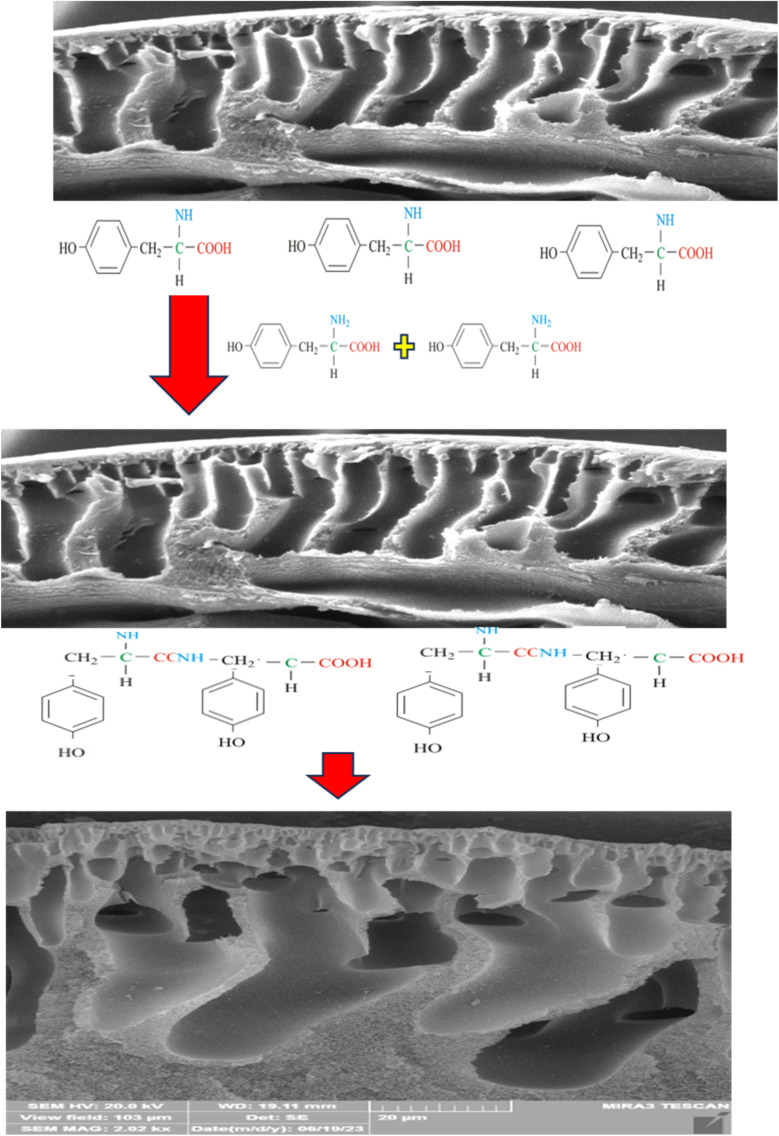
Proposed mechanism for the modification of PAN/GO HF using tyrosine.

Quasi-amide bonds may be formed as a result of the interaction of epoxy groups on the GO surface with the carboxyl groups of tyrosine. The peak at about 1640 cm^−1^ in the FTIR spectrum shows the formation of amide bonds. The peak at about 1735 cm^−1^, which is related to the stretching vibrations of the carbonyl group, also supports the presence of amide bonds on the surface. These peaks are visible especially in the surface-modified conditions with phosphate and amino acid and show that surface modification not only leads to the formation of amide bonds but also improves the surface properties and increases the system efficiency. It seems that phosphate acted as a linking agent in this process, has a catalytic effect and facilitates chemical reactions between carboxyl and epoxy or hydroxyl groups (Scheme 1). In 2015, Kim *et al.*, in a study investigating cobalt phosphate catalysts, showed that phosphates can be effective catalysts by adjusting the surface conditions and creating a suitable environment for chemical reactions.^36^ Especially in modified systems such as PAN/GO, phosphates can provide a more stable environment for chemical bonds. This is done by strengthening the stability of active groups and facilitating surface reactions, while the presence of phosphates is used to adjust the pH. These properties are especially important in processes that are sensitive to surface changes, such as adsorption or chemical reactions. For example, in some researches, it has been shown that amide bonds between amino acids and carbon materials can increase the adsorption of molecules on the surface and thus increase the catalytic activity.^37^

Therefore, tyrosine could significantly modify the surface properties of PAN fibers. According to the findings, adding tyrosine to the surface of hollow fibers enhanced the hydrophilicity of the surface and made it easier for water and metal ions to be adsorbed because the created active functional groups on hollow fiber can interact with metal ions in particular ways, thus promoting selective adsorption and improving the adsorption capacity. Thus, tyrosine can greatly increase the efficiency of PAN in water purification and metal ion extraction procedures.

### 3.7 Adsorption kinetic consideration

Chemical kinetics deals with the quantitative study of changes in pressure or concentration of reactant(s) with time in a chemical reaction. The process of surface adsorption is the absorption of atoms or molecules of liquid or gas in contact with the solid surface. In this process, molecules of the dissolved substance in one phase are drawn towards the interface with the other phase and accumulate, and the interaction between the solid phase (adsorbent) and the liquid phase of the solvent and solutes occurs. Therefore, kinetic studies on the adsorbent surface provide useful information about the adsorption rate of metal ions to investigate the adsorption behavior of metal ions during the extraction process. It can also facilitate the optimization of extraction process parameters to achieve efficiency. Hence, by investigating the kinetics of adsorption, it is feasible to improve the effectiveness of the extraction process, which saves time and resources.

**Fig. 7:**
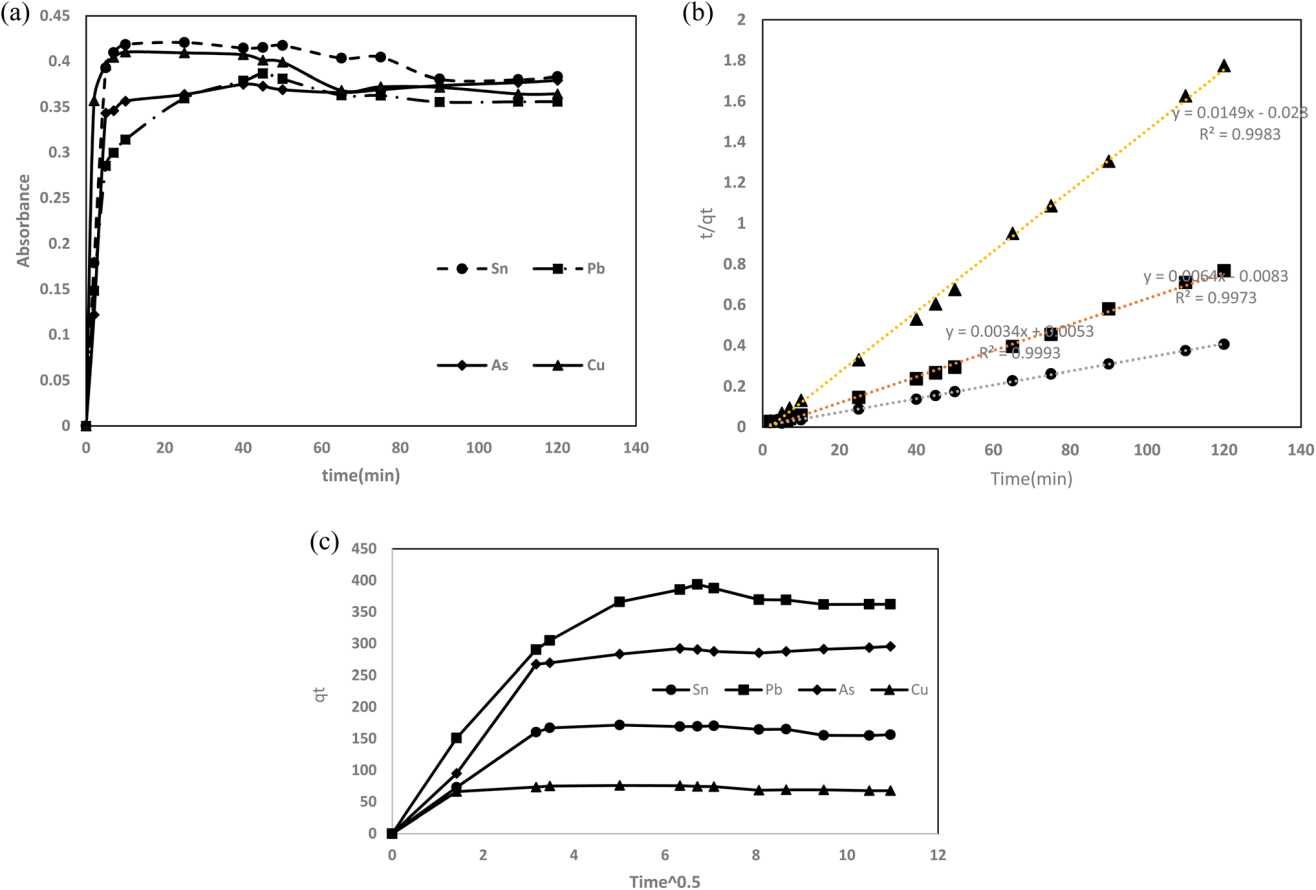
(a) Adsorption kinetics of metal ions during 140 min at optimum conditions, (b) pseudo-second order fitting of kinetic data and (c) intra-particle diffusion models employing kinetic adsorption of metal ions using PAN/GO-Tyr HF.

The adsorption kinetics of metal ions for determining an effective adsorption kinetic model were studied. Based on the adsorption profiles of metal ions given in Fig. 7a, the abundant active adsorption site on the surface of the PAN/GO-Tyr hollow fiber is thought to be responsible for the quick absorption of metal ions during the early stages of extraction. The number of active sites decrease throughout the adsorption process. Consequently, the uptake curves always plateau with time. In this study, to analyze the adsorption process and identify an appropriate adsorption kinetic model, quasi-first-order kinetic eqn (1) and (2) and pseudo-second order kinetic eqn (3) were used to fit and analyze the adsorption process.

4 ln(*q*_e_ − *q*_t_) = ln_*q*_e__ − *k*_1_*t*

Or5
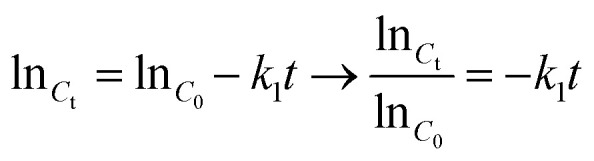
6
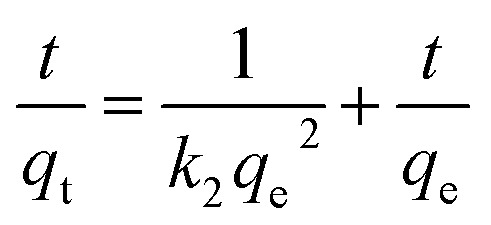


The adsorption capacity (*q*_t_) and equilibrium adsorption quantity (*q*_e_) are the two variables in the formula, and the rate constants of the quasi-first-order and quasi-second-order dynamic equations, respectively, are denoted by *k*_1_ (min^−1^) and *k*_2_ (g mg^−1^ min^−1^). The quasi-first-order equation's fitting curve is obtained by the ln(*q*_e_ − *q*_t_) *vs. t* plot (Fig. S5†), while the pseudo-second-order equation's fitting curve is obtained by the *t*/*q*_t_ plot. It is evident from Fig. S3† that a pseudo-first order model was not used to match the data. The correlation coefficient (*r*^2^) for model fitting was less than 0.8. It is evident from Fig. 7b that the adsorption kinetic data of the fabricated nanocomposite has been best fitted to the pseudo-second order kinetic equations and correlation coefficients of 0.9993, 0.9983, 0.9973, and 0.9973 for the As^3+^, Cu^2+^, Pb^2+^, and Sn^2+^ ions, respectively. The basic concept is that chemisorption is the rate-limiting step. The adsorption capacity of active sites is assumed to stay constant throughout the process by the model and there are no significant changes in the total number of accessible sites.

A diffusion-controlled exchange is usually considered in the adsorption of solutes from a solution. According to the results, the pseudo-second order model is more dominant and it can be said that chemical adsorption occurs in several stages between the metal ions and the PAN/GO-Tyr adsorbent through electrostatic interactions.^38^

The intra-particle diffusion model was additionally utilized to investigate the metal ion diffusion process *via* the adsorbent in a kinetic simulation. Intra-particle diffusion is an important aspect of solid particle membrane extraction (SPME), especially when hollow fibers are used as the extraction medium. In this study, the fibers were also modified by the amino acid tyrosine. Analytes are expected to be adsorbed from the sample matrix onto the surface of the coating, but for complete extraction, they must also diffuse into the fibers. The model is a straightforward approximation of pore diffusion kinetics and could be used to determine the diffusion mechanism that controlled sorption.^39^

Also, intra-particle diffusion models employing the following equation were considered.

7 *q*_t_ = *k*_diff_*t*^1/2^ + *C*

In the above equation, *k*_diff_ (mg g^−1^ min^−1^) and C are intra-particle diffusion rate constant and mass transfer resistance constant in the boundary layer (refers to the thickness of the boundary layer), respectively.

The curves obtained at various periods show multi-linearity, as shown in Fig. 7c. Multi-linearity reveals that the adsorption process of metal ions onto PAN/GO-Tyr HF surface follows a film diffusion pattern and has many rate-limiting phases, confirming that the adsorption mechanism comprises of various steps. Table 5 displays the intra-particle diffusion parameter (*k*_id_), which was calculated using the slope of *q*_t_ against square root of time plot and additional relevant factors. According to these results, the first linear segment typically represents the initial stage of the process, where rapid adsorption or extraction occurs. During 0–15 minutes, the analytes are rapidly transported from the sample matrix to the extraction phase coating, usually due to external mass transfer processes. This stage is characterized by high rates of adsorption or extraction. The second part is the adsorption period (20–50 minutes), representing a pattern from the adsorbate to the active sites of each adsorbent through interactions with the active functional groups of the adsorbent. Since intra-particle diffusion is the main rate-controlling step, the second linear section does not pass through the origin (*C* ≠ 0), indicating that external mass transfer also occurs concurrently.^40,41^ The third linear segment, occurring at higher times, typically represents an equilibrium-controlled or diffusion-controlled stage. At this stage, the rate of adsorption or extraction slows down because the system approaches equilibrium because analyte molecules can penetrate deeper layers or pores of the adsorbent. In other words, the rate of absorption is affected by intraparticle diffusion processes. Thus, first, rapid adsorption occurred on the external surface, and with the continuation of slow adsorption inside the particles, the equilibrium was reached. In other words, the process of adsorbing ions by the membranes was generally controlled by chemisorption and completed by intraparticle diffusion.

**Table 5:** Intra-particle diffusion parameter (*k*_id_), times and *r*^2^ values for different segments of the plot *q*_t_*versus* square root of time

Metal ion	Step 1			Step 2			Step 3		
	*r* ^2^	*k*_id_	Time interval (min)	*r* ^2^	Kid	Time interval (min)	*r* ^2^	*k*_id_	Time interval (min)
As^3+^	0.993	151.6	0–10	1	14.68	15–50	0.990	3.6	60–120
Sn^2+^	0.999	50.63	0–10	0.505	0.882	20–50	0.715	3.55	60–120
Pb^2+^	0.940	131.54	0–10	0.861	12.30	20–50	0.9909	2.81	60–120
Cu^2+^	0.975	7.368	0–10	0.833	0.796	20–50	0.548	0.401	60–120

## 4. Conclusion

Despite having advantages such as high mechanical strength, thermal and chemical stability, and high adsorption performance, restrictions such as inappropriate selectivity, poor hydrophilicity, and low regeneration efficiency in chemisorption limit the practical performance of PAN membranes. Herein, GO doping in PAN structure and surface modification using tyrosine (Tyr) were used. PAN/GO-Tyr fiber membrane was successfully prepared by phase inversion of PAN solution with GO nanoparticles and subsequent self-polymerization of Tyr on PAN/GO HF membranes. This is a first time that PAN/GO hollow fiber surface was chemically modified and a dual layer hollow fiber was created. Also, tyrosine, as a natural and bio-compatible ligand, was used for the monitoring of removal and adsorption of metal ions in water, with very low detection limits obtained for ions. Surface characterization revealed the successful synthesis of hollow fiber and the modification of the surface by tyrosine, which led to significant changes in the morphology, creating a smooth surface and mesoporous surface. Furthermore, due to PAN/GO-Tyr HF's quick adsorption kinetics, it reached equilibrium in about 20 minutes, supporting its potential for quick and effective treatment, which would reduce treatment time and related energy consumption. The fitting of the pseudo-second-order for the kinetics highlights a robust adsorption mechanism involving multilayer adsorption and chemisorption processes. These advantages make PAN/GO-Tyr hollow fiber a promising material for various environmental and industrial applications requiring efficient metal ion removal with specific purposes, such as enhancing the selectivity and adsorption capacity and faster kinetics. According to the primary goal of WHO, which is to identify toxic ions in solutions, we have introduced a sensitive extraction–spectrophotometric ultrasensitive method for the detection of metal ions in water samples. The detection limits of As^3+^, Cu^2+^, Sn^2+^, and Pb^2+^ were computed to be 0.004, 0.005, 0.002, and 0.01, respectively. These results were superior to most published values in the literature and below the safety limit in tap water. Another benefit is the ability to pre-concentrate several target ions and their determination across a broad linear dynamic range. Thus, PAN/GO-Tyr HF may be used to treat target ions present in water sources in an efficient manner.

## Data availability

All the data have been included in the manuscript.

## Author contributions

M. A. T.: supervision, project administration, methodology, software, validation, investigation. S. A. H.: methodology, validation, G. A.: resources, data curation, F.·B.: methodology, software, validation, data curation.

## Ethical approval

This article does not contain any studies with human participants or animals performed by any of the authors. Therefore, as an observational study, it doesn't require any ethical approval.

## Conflicts of interest

The authors declare no conflict of interest.

## Supplementary Material

RA-015-D4RA08423C-s001
