# Supplementary material for: Development and characterization of PAN/GO-tyrosine hollow fiber membranes for enhanced heavy metal adsorption and SPME-spectrophotometric detection
Source: RSC Adv. 2025 Feb 5;15(5):3721–37. doi: 10.1039/d4ra08423c (PMC11797155; doi:10.1039/d4ra08423c)
Supplement: RA-015-D4RA08423C-s001 [file RA-015-D4RA08423C-s001.pdf]

Supplementary Information (SI) for RSC Advances.
This journal is © The Royal Society of Chemistry 2025

Development and Characterization of PAN/GO-Tyrosine Hollow Fiber Membranes for Enhanced Heavy Metal Adsorption and SPME-Spectrophotometric Detection

Maryam Abbasi Tarighat*[1], Fatemeh Barghandan[1], Seyed Abdollatif Hashemifard[2], Gholamreza Abdi[3]

[1]Deparmen of Chemistry, Faculty of Nano and Bio Science and Technology, Persian Gulf University, Bushehr 75169, Iran

[2]Sustainable Membrane Technology Research Group, Faculty of Petroleum, Gas and Petrochemical Engineering, Persian Gulf University, Bushehr 75169, Iran

[3]Department of Biotechnology, Persian Gulf Research Institute, Persian Gulf University, Bushehr, 75169, Iran

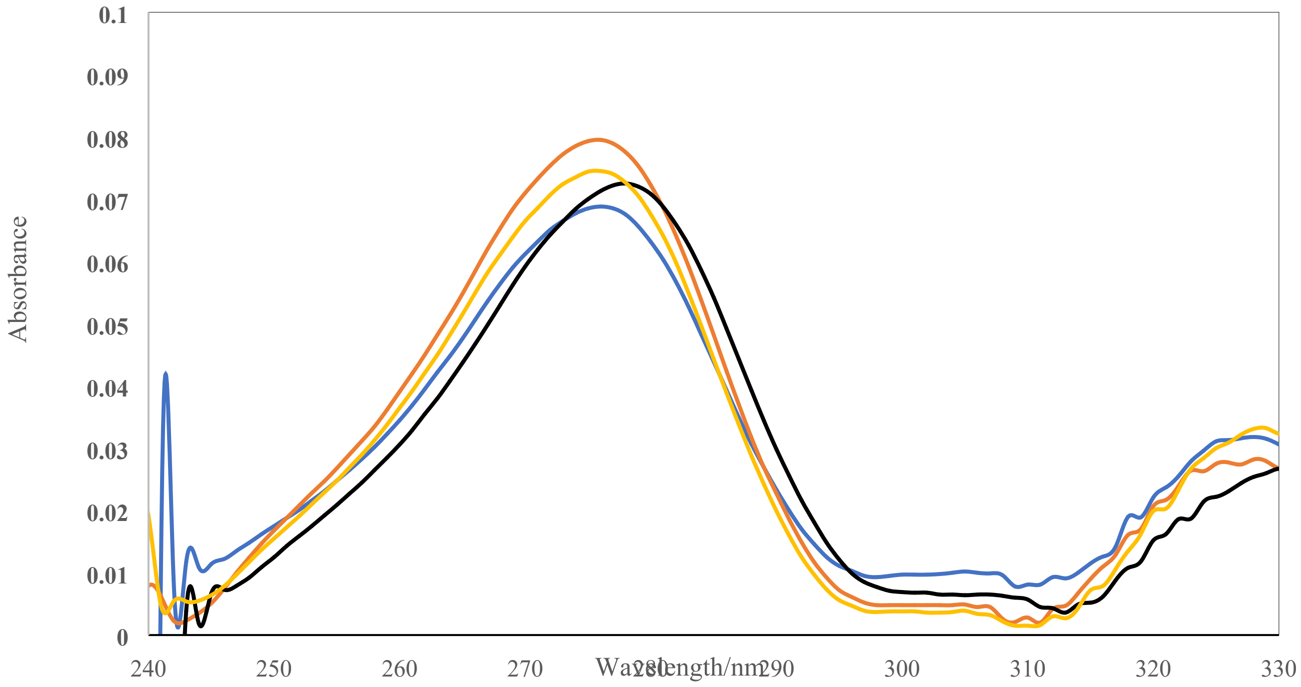

Fig. 1S. Absorbance spectra of metal ions As^{3+}(blue line), Pb^{2+}(orange line), Sn^{2+}(red line) and Cu^{2+} (black line) using PAN/GO Hollow fiber

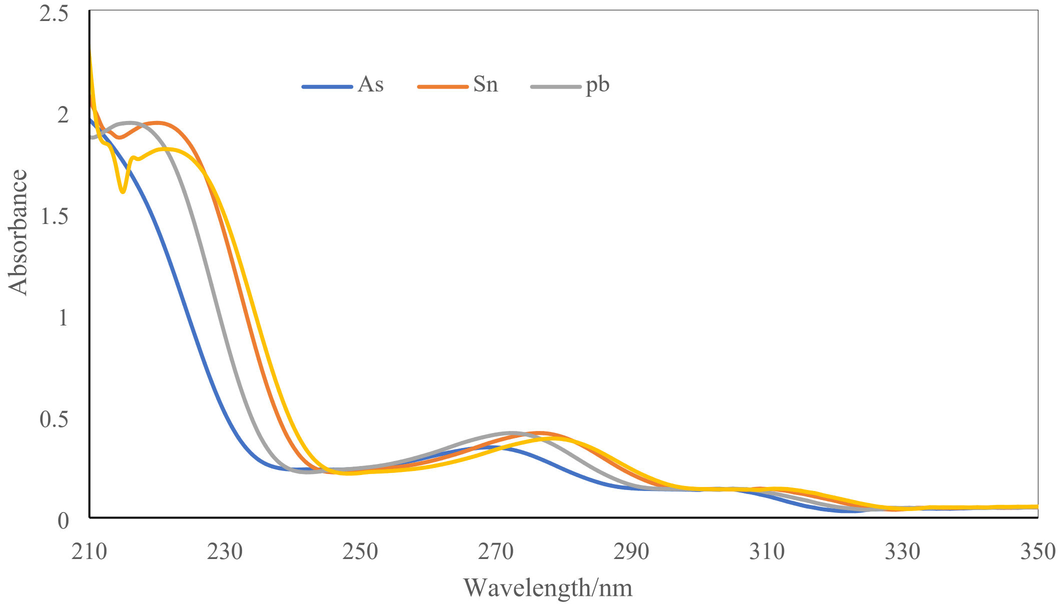

Fig. 2S. UV-Vis spectra of metal ions with tyrosine as a ligand

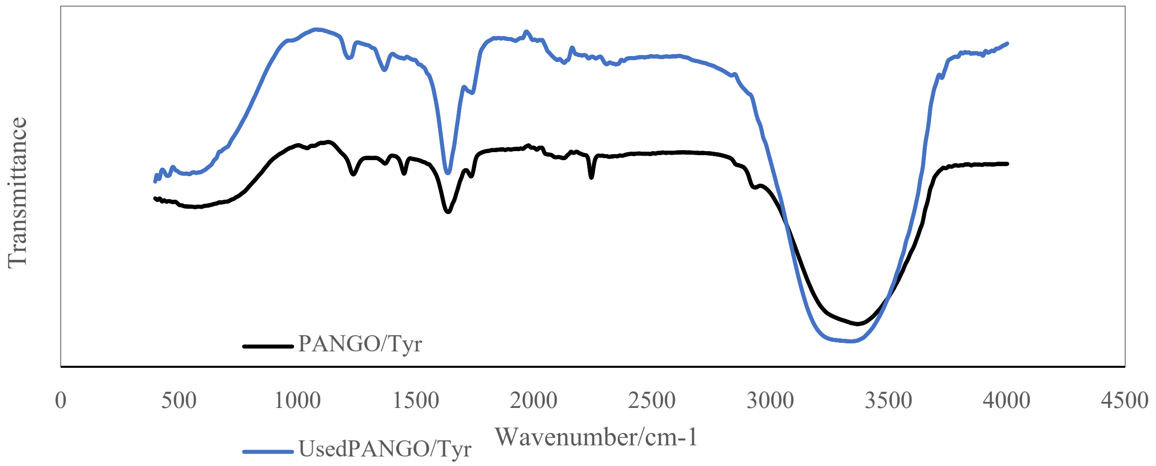

Fig. 3S. FTIR spectra of PAN/GO-Tyr and used PAN/Go-Tyr after 6 cycles

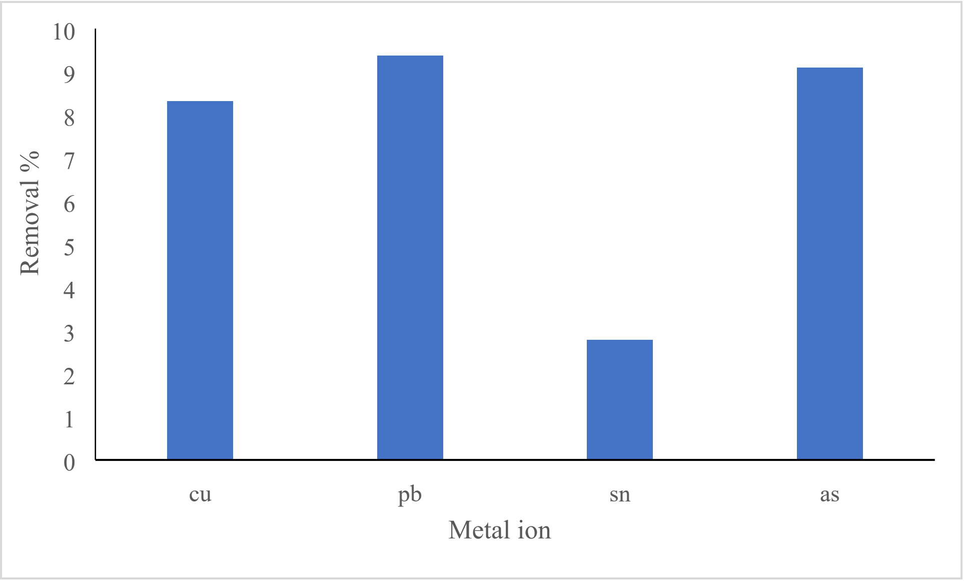

Fig. 4S. Removal percentage of metal ions after 6 cycles

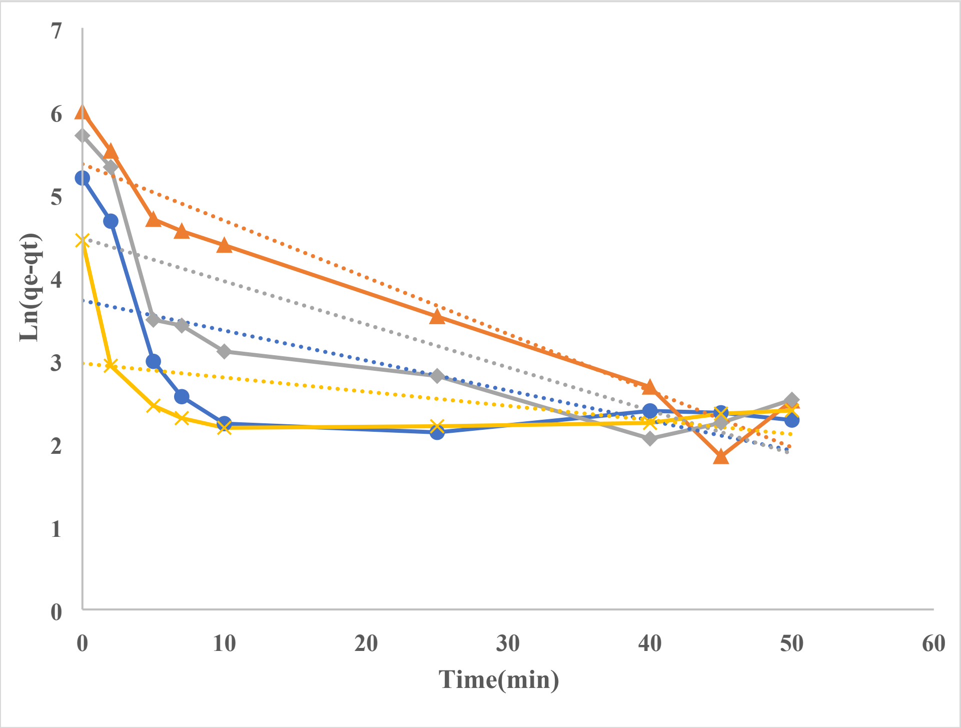

Fig. 5S Fitting the kinetic data obtained for As3+(▪), Sn2+(●), Pb2+(▲) and Cu2+(×) using pseudo-firstorder, Model
